# Perceived social support and depressive symptoms among vocational college students: the serial mediating roles of positive coping and post-stress growth

**DOI:** 10.3389/fpsyg.2025.1694285

**Published:** 2026-01-08

**Authors:** Wenfang Lu, Mingkun Ouyang, Xiaoyu Huang

**Affiliations:** 1 Guangxi Eco-Engineering Vocational and Technical College, Liuzhou, China; 2 College of Education Science, Guangxi Minzu University, Nanning, China

**Keywords:** vocational college students, perceived social support, positive coping strategies, post-stress growth, depression

## Abstract

Vocational college students face heightened depression risks due to unique psychosocial stressors, yet mechanisms linking perceived social support to depression mitigation remain inadequately explored. This cross-sectional study investigated the serial mediating roles of positive coping strategies and post-stress growth in 960 Chinese vocational students using validated scales (perceived social support scale, simplified coping style questionnaire, post-stress growth inventory, self-rating depression scale) analyzed via Hayes’ PROCESS Model 6. Results demonstrated that perceived social support significantly reduced depressive symptoms through three pathways: direct effects, independent mediation via positive coping strategies, independent mediation through post-stress growth, and sequential mediation where support enhanced coping strategies that subsequently fostered post-stress growth and reduced depression. Collectively, these adaptive psychological processes accounted for the majority of the total protective effect. Findings underscore the value of multipathway campus interventions targeting social support networks, cognitive-behavioral coping training, and growth-focused resilience programs to address mental health disparities in vocational education populations.

## 1 Introduction

In recent years, depressive symptoms among college students have exhibited a concerning upward trajectory, which has garnered significant scholarly and societal concern. A nationwide meta-analysis revealed that 28.4% of Chinese university students exhibit clinically relevant depressive manifestations (Gao et al., 2020). Higher vocational colleges, serving as the primary base for cultivating highly skilled professionals in China, bear the dual mandate of imparting professional skills and nurturing well-rounded individuals with sound physical and mental health. Psychological wellness education, as a critical component of China’s higher education system, directly impacts students’ psychological resilience, physical and mental well-being, and the stable development of society. According to the 2024 Statistical Bulletin on China’s Education Development, China had 1,613 higher vocational colleges by 2024, with an enrollment exceeding 17 million students, accounting for a substantial proportion of the total tertiary student population. However, the psychosocial challenges faced by vocational college students, who demonstrate greater vulnerability to mental health disorders than their academic university counterparts do, are particularly noteworthy (Yu et al., 2017). This disparity stems from multiple systemic factors, including institutional-level psychological support deficiencies and population-specific resilience limitations, which results in an elevated depression risk for vocational education populations (Coledam et al., 2022; He et al., 2023; Zhang et al., 2023). Of particular concern is the established association of depression with the increased risks of suicidal ideation and maladaptive behavioral patterns, which underscores the profound personal and social ramifications of depression (Benatov et al., 2021; Sarason et al., 1991). These epidemiological realities call for rigorous investigations into the relevant etiological mechanisms and protective pathways to inform targeted intervention strategies for vocational college populations.

Perceived social support serves as a critical protective factor against depressive mood in vocational college populations. Defined as individuals’ subjective appraisals concerning being valued, understood, and assisted within their social networks (Sarason et al., 1991) this construct operates through dual mechanisms that are articulated by the social convoy model and main-effect model (Cohen and Wills, 1985; Peek and Lin, 1999). First, perceived social support facilitates the cultivation of adaptive psychological resources such as hopefulness and emotional resilience (Yuan et al., 2018), which function as buffers against psychopathological outcomes (Hua and Ma, 2022). Second, this construct provides tangible and affective support during adversity through tripartite support systems (parental, academic, and peer networks), thereby mitigating depression risk (Glickman et al., 2021; Liu et al., 2023).

Empirical evidence from diverse populations consistently corroborates that perceived social support mitigates depressive symptoms (Rowland et al., 2023; Shah et al., 2024; Tilahune et al., 2022). However, the extant research has been focused predominantly on nonvocational cohorts, including adolescents (Gariepy et al., 2016; Letkiewicz et al., 2023) and traditional undergraduates (Wang et al., 2024; Yu et al., 2023) creating a critical evidence gap in regard to vocational education contexts. This methodological oversight necessitates the population-specific verification of the depression-alleviating effects of social support. Grounded in theoretical frameworks and cross-population findings, we hypothesize a significant negative association between perceived social support and depressive symptoms among vocational college students. Furthermore, according to the indirect effect model of social support, social support indirectly affects an individual’s mental health through other factors (Liu et al., 2023; Xie et al., 2024). However, the previous research into the ways that perceived social support affects depression among vocational college students is incomplete. Thus, the aim of this study is to investigate the chained mediating effects of positive coping strategies and post-stress growth on the relationship between perceived social support and depression among vocational college students.

### 1.1 Mediating role of positive coping strategies

Positive coping strategies, which are defined as adaptive behavioral patterns involving support seeking and cognitive reframing in the face of stressors (Skinner et al., 2003), act as pivotal mediators in psychopathological processes. The cognitive-transactional process theory (CPT) of stress (Gee, 2008) posits that coping mechanisms interact with contextual resources to determine mental health outcomes. Specifically, enhanced psychosocial assets, particularly robust social support networks, catalyze the activation of positive coping repertoires, thereby facilitating problem resolution and developmental growth while attenuating depressive symptomatology (Hou et al., 2024; Liu et al., 2023; Yang et al., 2022). This mechanism has been corroborated by meta-analytic evidence that demonstrates significant associations among social support, adaptive coping, and reduced depression severity (Li et al., 2012).

Crucially, recent mechanistic studies have identified positive coping strategies as transduction channels through which social support exerts mental health benefits. Empirical data have revealed that perceived support adequacy (a) enhances self-efficacy beliefs in stress management and (b) provides coping modeling templates through social learning, which collectively increases the likelihood of adopting adaptive strategies (Hao et al., 2023; Hou et al., 2024; Yang et al., 2022). For instance, comparative analyses have indicated that individuals with stronger support systems demonstrate a greater utilization of cognitive restructuring techniques during adversity than their less-supported counterparts do (Hou et al., 2024). This mediation pathway ultimately contributes to emotional regulation and depressive symptom reduction, and multiple studies have confirmed its statistical significance across diverse populations (Hao et al., 2023).

Good perceived social support is conducive to the adoption of positive coping strategies by individuals. Multiple studies have demonstrated a significant positive correlation between the level of perceived social support and that of positive coping strategies (Buitron et al., 2023). Perceived social support plays an important role in determining the coping strategies that are adopted by college students (Wang et al., 2023). Research shows that there is a significant positive correlation between perceived social support and positive coping strategies (Buitron et al., 2023). When an individual suffers from cyberbullying, perceived social support from parents, teachers, peers, etc., can provide that individual with strategies for coping with stress and negative emotions and encourage individuals to adopt effective coping strategies to overcome trouble (Espino et al., 2023). In addition, research has shown that positive coping strategies can negatively predict an individual’s depressive mood (Shi et al., 2023). That is, when an individual faces stress, they adjust their own cognition and behavior to adapt to changes in the external and internal environment (Lazarus and Folkman, 1984), thereby alleviating their depressive mood (Zhang and Hu, 2024). Although previous studies have provided much empirical evidence indicating that coping strategies play a mediating role between social support and depression, most studies have been mainly focused on nonvocational college student groups, particularly adolescents or other age groups (Cao et al., 2024; Liu et al., 2023; Wang et al., 2023). Therefore, this study takes vocational college students as the research objects with the aim of exploring the possible mediating role played by positive coping strategies between perceived social support and the depressive mood of vocational college students.

Robust perceived social support significantly predicts the adoption of positive coping strategies among college populations. Empirical evidence has consistently revealed a strong positive association between perceived support levels and adaptive coping engagement (Buitron et al., 2023; Wang et al., 2023). In particular, in vocational education contexts, perceived support acts as a critical determinant of student stress management approaches. When facing adversities such as cyberbullying, multisource support systems (parental, academic, and peer networks) are used to equip individuals with coping toolkits to facilitate effective emotional regulation and problem solving (Espino et al., 2023).

The depression-alleviating function of positive coping strategies is well established to function through bidirectional mechanisms: (1) Cognitive-behavioral adjustments enable individuals to reconcile their internal/external environmental demands (Lazarus and Folkman, 1984), and (2) proactive stress engagement disrupts maladaptive rumination cycles, thereby collectively reducing depressive symptomatology (Shi et al., 2023; Zhang and Hu, 2024). Although the literature confirms the mediating role of coping strategies in the social support–depression nexus (Cao et al., 2024; Liu et al., 2023), some notable sampling limitations persist. Current investigations are predominantly focused on nonvocational cohorts, adolescents and traditional undergraduates in particular, leaving the psychological dynamics of vocational students underexplored (Wang et al., 2023).

This study addresses this critical evidence gap through a specific examination of vocational college students. Building upon the cognitive-transactional process theory framework, we propose the following:

*H1:* Positive coping strategies mediate the protective effects of perceived social support against depressive mood among vocational college students.

The findings are aimed at informing the development of context-sensitive interventions tailored to vocational education environments.

### 1.2 The mediating role of post-stress growth

Post-stress growth refers to the multidimensional psychological adaptation through which individuals attain increased existential awareness and maturation following exposure to adversity (Hu et al., 2021). Grounded in the posttraumatic growth model (PTG) (Tedeschi and Calhoun, 2004), this construct emerges from social-cognitive processing, wherein external support systems facilitate the following: (1) affective scaffolding, which provides safe spaces for emotional disclosure; and (2) meaning-making incubation, which involves reframing stressors as developmental catalysts. Empirical evidence has demonstrated that social support alleviates trauma-related cognitive dissonance through resource provision, activates benefit-finding schemas via guided cognitive reappraisal, and promotes the narrative reconstruction of adverse experiences (Wang and Wu, 2020). Thus, perceived social support is a pivotal antecedent of post-stress growth, and it operates through tripartite mechanisms comprising emotional validation, coping model demonstration, and existential meaning coconstruction. Empirical investigations have consistently demonstrated a significant positive association between perceived social support and post-stress growth trajectories (Grover et al., 2024; Liu et al., 2021; Nik Jaafar et al., 2022; Zhang et al., 2022b). This protective mechanism operates through two pathways: (1) psychosocial safety perceptions, in which heightened support fosters environmental security and thereby reduces hypervigilance to threats, and (2) strategic resource mobilization, in which support networks provide tangible coping blueprints and informational capital to enhance stress adaptation. Individuals with elevated social support demonstrate significantly greater growth attainment following adversity, as they can more effectively reframe challenges as developmental opportunities (Zhang et al., 2022b). Emerging evidence suggests a moderated pathway wherein post-stress growth modulates the social support–depression nexus. The dynamic adaptation model posits that while adversity exposure can initially increase the risk of depression, the activation of internal protective mechanisms (e.g., cognitive reappraisal, meaning reconstruction, etc.) during stress processing can attenuate psychopathological outcomes. Specifically, when perceived social support triggers adaptive cognitive processing, such as the reframing of stressors as developmental challenges, it initiates benefit-finding mechanisms to counteract depressive symptomatology. This salutogenic cascade explains how individuals with equivalent support levels can exhibit differential depression vulnerability on the basis of their capacity for post-stress growth. Notably, the emerging research positions post-stress growth as a critical resilience-building mechanism that simultaneously attenuates psychopathological risk and enhances psychological health (Casali et al., 2022; Carr, 2007). These empirical observations corroborate the hypothesized dual-function of post-stress growth: (1) protective buffering to mitigate the neurocognitive impact of chronic stress and (2) developmental potentiation to foster meta-cognitive skills for the prevention of future adversity. These findings underscore the necessity of integrating growth-oriented paradigms into mental health interventions for vocational students. Successful adversity resolution facilitates multidimensional psychological development—including the strengthening of cognitive flexibility, emotional resilience, and self-efficacy—which serves as a critical determinant of long-term mental well-being. Convergent empirical evidence has established post-stress growth as a robust negative predictor of depressive symptomology, with higher growth levels correlating with a reduction in clinical depression risk (Boehm-Tabib and Gelkopf, 2021; Park and Im, 2021). This process indicates that the successful resolution of stressful events through active efforts leads to beneficial cognitive transformations that enable individuals to restore their psychological equilibrium. Simultaneously, this experience fosters psychological maturation through adaptive growth, which manifests in cognitive strategy optimization (e.g., the reframing of stressors as manageable challenges) and positive emotional enhancement (e.g., an increase in hopefulness and self-efficacy). These synergistic changes collectively attenuate depressive symptoms by addressing both cognitive appraisals and affective adversity responses. Research has predominantly conceptualized post-stress growth as an endpoint phenomenon (Hu et al., 2021; Lo et al., 2023; Zhang et al., 2022a) and has engaged in limited exploration of its mediatory functions in psychopathological processes. To address this critical gap, we formally hypothesize the following:

*H2:* Post-stress growth mediates the relationship between perceived social support and depressive mood among vocational college students.

This investigation is meant to extend prior work by systematically examining stress-induced growth as a dynamic mediator and testing its role in in the protective effects of social support in vocational education contexts.

### 1.3 Chain-mediating role of positive coping strategies and post-stress growth

In addition to their independent mediating effects, positive coping strategies and post-stress growth can sequentially mediate the relationship between perceived social support and depressive mood among vocational college students. The posttraumatic growth model (Tedeschi and Calhoun, 1996) provides a theoretical foundation for this chained mechanism, proposing that adversity resolution fosters psychological development through three interconnected domains: the enhancement of self-awareness, the strengthening of interpersonal relationships, and the deepening of philosophical perspectives on life. This theoretical framework suggests that adaptive coping efforts (e.g., proactive problem solving) might serve as initial catalysts for growth processes, which subsequently contribute to emotional regulation and symptom reduction. Specifically, exposure to adverse life events facilitates tripartite psychological adaptation, which involves the deepening of self-awareness through introspective processing, the strengthening of interpersonal competence via increased support-seeking behaviors, and the cultivating of existential wisdom through meaning reconstruction. This process aligns with established growth models wherein adversity serves as a catalyst for cognitive–behavioral maturation. These adaptive transformations align with the core premise of the posttraumatic growth model, suggesting that adversity exposure, when channeled through effective coping strategies, can paradoxically enhance psychological functioning. Empirical evidence has further established that post-stress growth development is contingent upon two key psychosocial resources: the availability of perceived social support and the implementation of positive coping methods (Zhang et al., 2022b). In support of this model, longitudinal investigations have demonstrated that proactive coping behaviors predict subsequent growth attainment following adversity (Peters et al., 2021; Wan et al., 2022). Collectively, these findings underscore the necessity of examining coping-growth dynamics in vocational education contexts. The implementation of positive coping strategies following adversity exposure facilitates the strategic mobilization of socially derived resources, thereby enabling effective problem navigation while fostering psychological maturation (Boehm-Tabib and Gelkopf, 2021; Park and Im, 2021). Furthermore, the developmental trajectory of post-stress growth amplifies adaptive cognitive-affective processing—specifically through the enhancement of meaning reconstruction and emotional regulation capacity—which establishes resilience pathways for depressive symptom mitigation. This dynamic interplay between coping efforts and growth attainment highlights the phased progression of adaptive stress response mechanisms. While the extant research has established perceived social support (Park, 2024), positive coping strategies (Han et al., 2023), and post-stress growth (Zhong et al., 2024) as key protective factors against adolescent depression, the specific mechanisms through which these psychosocial resources interact within vocational education populations remain underexplored. To address this critical gap, we propose the following:

*H3:* Positive coping strategies and post-stress growth sequentially mediate the association between perceived social support and depressive mood among vocational college students.

This investigation is meant to systematically examine the temporal mediation pathway whereby social support first enhances adaptive coping, which subsequently facilitates stress-induced growth and ultimately reduces depression risk.

### 1.4 Study objectives and significance

The extant literature substantiates depression as a pivotal psychosocial challenge that impedes the mental health development of vocational college students. Despite this recognition, critical knowledge gaps persist within this research domain. The current investigations remain disproportionately focused on adolescent and traditional undergraduate populations and largely overlook the unique stressors and developmental trajectories that are characteristic of vocational education contexts. Furthermore, the existing mechanistic models frequently take reductionist approaches by examining singular mediators, either positive coping strategies or post-stress growth, thereby neglecting the potential synergistic interplay that occurs between them in adaptation processes. The paucity of translational research that bridges theoretical insights to targeted interventions compounds these limitations for this vulnerable population.

To address these interrelated shortcomings, the present study includes two primary objectives. The first of these is to elucidate the chained mediation mechanisms through which perceived social support alleviates depressive mood via sequential enhancements of positive coping strategies and post-stress growth among vocational college students. The second aim is to identify actionable intervention targets by delineating modifiable psychosocial pathways within vocational education ecosystems. Grounded in the posttraumatic growth model and stress-coping theories, this investigation contributes dual advancements to the field: it offers theoretical advancement by modeling multistage adaptation processes in vocational populations and practical advancement by informing the design of context-sensitive mental health programs meant to concurrently strengthen social support networks, cultivate adaptive coping repertoires, and facilitate postadversity maturation.

## 2 Materials and methods

### 2.1 Participants and procedure

A convenient sampling method was used to select 960 students from three higher vocational colleges in Guangxi, Guangxi, Guangxi, China. After eliminating invalid questionnaires, 921 valid questionnaires (95.9%) were obtained. Following rigorous data screening meant to exclude incomplete or inconsistent responses (*n* = 39), the final analytical sample comprised 921 students (retention rate = 95.9%). The demographic characteristics of these students include a gender distribution of 592 males (64.3%) and 329 females (35.7%) and an academic standing distribution of 554 freshmen (60.1%), 301 sophomores (32.7%), and 66 juniors (7.2%). The cohort exhibited significant rural–urban disparities, with 777 participants (84.4%) living in rural areas versus 144 urban residents (15.6%). The family composition data revealed 122 only children (13.2%) and 799 siblings (86.8%).

A standardized survey was conducted by trained research assistants during class sessions between September and October 2023. Standardized protocols included (1) a verbal briefing detailing the study objectives and offering confidentiality assurances, (2) the distribution of consent forms emphasizing voluntary participation, and (3) the monitoring of completion sessions to ensure data integrity. All procedures received ethical approval from the institutional review board and adhere to the Declaration of Helsinki guidelines for human subject research.

### 2.2 Measures

#### 2.2.1 Perceived social support

The participants’ levels of perceived social support were assessed using a culturally adapted Chinese version of the perceived social support scale (Blumenthal et al., 1987; Huang et al., 1996). This 12-item instrument is used to evaluate three distinct support domains: familial support (4 items), peer support (4 items), and external social network support (4 items). The respondents rated their level of agreement on a 7-point Likert scale (1 = strongly disagree to 7 = strongly agree), with higher composite scores indicating greater levels of perceived support availability. The measure demonstrated excellent reliability in our sample (Cronbach’s *α* = 0.935), which aligns with previous validation studies conducted among Chinese populations.

#### 2.2.2 Positive coping strategies

The positive coping subscale from Xie’s (1998) simplified coping style questionnaire was employed to assess adaptive stress management behaviors. This 10-item instrument is used to measure proactive engagement strategies across cognitive (e.g., “I try to see problems as challenges”) and behavioral (e.g., “I seek advice from others when facing difficulties”) domains. The participants rated their typical response frequency using a 4-point Likert scale (1 = never to 4 = always), with higher scores indicating a greater utilization of constructive coping methods. The subscale demonstrated excellent internal consistency in this study (*α* = 0.898), which is in alignment with its established psychometric properties for Chinese student populations.

#### 2.2.3 Post-stress growth

Post-stress growth and post adversity developmental outcomes were measured using the post-stress growth inventory for college students, which is a culturally validated Chinese instrument developed by Wu and Fang (2014). The 18-item scale is used to assess the following four theoretical dimensions: existential perspectives (the reappraisal of life values), relational competence (enhanced interpersonal skills), coping mastery (effective stress management strategies), and metacognitive awareness (the refinement of self-concept). The participants rated their post-stress adaptation experiences using a 4-point Likert scale (0 = not at all to 3 = very much), where higher composite scores indicate greater psychological growth following adversity. This measure demonstrated exceptional reliability in the current sample (Cronbach’s α = 0.948), which aligns with its robust psychometric validation that occurred in prior studies on Chinese college populations.

##### 2.2.3.1 Depressive symptoms

Depressive symptom severity was assessed using self-rating depression scale (SDS), which is a widely validated 20-item self-report measure. The participants rated the frequency of affective, psychological, and somatic symptoms over the preceding week using a 4-point Likert scale (1 = a little of the time to 4 = most of the time). The raw scores were converted to standardized scores by multiplying the total score by 1.25 and retaining the integer value, with higher final scores indicating greater depression severity. The SDS demonstrated good internal consistency in this sample (α = 0.821), which is in alignment with its established reliability across diverse clinical and nonclinical populations.

### 2.3 Statistical processing

The statistical analyses for this study were conducted using SPSS 26.0 software in conjunction with the PROCESS 4.0 macro program (Hayes, 2017). To address potential common method bias, we implemented Harman’s single-factor test following the methodological guidelines established in previous research. Exploratory factor analysis revealed nine factors with eigenvalues in excess of 1.0, with the first unrotated factor accounting for 24.628% of the total variance. This percentage remained below the recommended threshold of 40%, suggesting that common method variance did not substantially influence the study results. The analytical approach ensured methodological rigor through the adoption of appropriate statistical controls and verification procedures.

## 3 Results

### 3.1 Statistical analysis of demographic variable differences

Based on collected student demographic data, this study used independent samples t-tests to examine differences in perceived social support, positive coping style, post-stress growth, and depressive symptoms across gender, household registration (urban vs. rural), and only-child status groups. The analysis revealed no statistically significant differences (all *p* > 0.05) in any variable scores between urban and rural groups or between only-child and non-only-child groups. However, a significant gender difference emerged in depressive symptoms (t = −2.332, *p* < 0.001), with female students scoring significantly higher than males. No significant gender differences were observed in perceived social support, positive coping style, or post-traumatic growth (all *p* > 0.05). Detailed results are presented in Table 1.

**Table 1:** Statistical analysis of demographic variable differences.

Item	Gender (*M* ± *SD*)			Place of origin (*M* ± *SD*)			Only child (*M* ± *SD*)		
	Male (*n* = 592)	Female (*n* = 329)	*t*	Urban (*n* = 144)	Rural (*n* = 777)	t	Yes (*n* = 122)	No (*n* = 799)	*t*
Perceived social support	60.13 ± 11.90	59.00 ± 10.08	1.418	59.49 ± 11.69	59.77 ± 11.50	−0.271	58.49 ± 11.45	59.91 ± 11.54	−1.269
Positive coping	33.91 ± 7.04	33.48 ± 6.72	0.886	33.40 ± 7.34	33.82 ± 6.85	−0.663	33.67 ± 7.02	33.77 ± 6.92	−0.141
Post-Stress growth	69.34 ± 11.15	68.30 ± 10.50	1.376	68.94 ± 12.41	68.97 ± 10.64	−0.037	67.94 ± 11.47	69.13 ± 10.84	−1.113
Depressive symptoms	41.40 ± 8.81	42.75 ± 7.74	−2.332***	42.14 ± 8.82	41.84 ± 8.40	0.394	41.70 ± 8.55	41.90 ± 8.45	−0.136

^*^p < 0.05. ^**^p < 0.01. ^***^p < 0.001.

### 3.2 Depression status of vocational college students

The SDS standardized score interpretations followed the established clinical thresholds of the Chinese norm (Wang and Wu, 2023), where scores below a value of 53 indicate a normative mental health status, those ranging from 53–62 reflect mild depressive symptoms, those ranging from 63–72 suggest moderate depression, and those in excess of 72 correspond to severe depression. Among the vocational college participants, depressive symptom severity manifested across four distinct tiers: nearly half of the sample (46.47%, *n* = 428) fell within the normative range, whereas a significant proportion presented mild (29.75%, *n* = 274) or moderate (22.04%, *n* = 203) symptom levels, and 1.74% of the respondents (*n* = 16) met the criteria for severe depression. These findings reveal a concerning pattern of depressive symptom prevalence within the vocational college student population, with over half of the participants demonstrating clinically meaningful levels of psychological distress.

### 3.3 Intervariable relationships among social support, coping strategies, post-stress growth, and depressive symptoms

As shown in Table 2, the Kolmogorov–Smirnov test indicated non-normal distribution for all variables (all *p* < 0.05), thus Spearman’s rank-order correlation analysis was employed. The results revealed significantly positive correlations among perceived social support, positive coping style, and post-stress growth (*r* = 0.502 to 0.522, *p* < 0.001); whereas all three variables showed significantly negative correlations with depressive mood (*r* = −0.445 to −0.405, *p* < 0.001) (Table 3).

**Table 2:** Results of the Kolmogorov–Smirnov normality test for key variables.

	Kolmogorov-Smirnov^a^		
	Statistic	df	Significance
Perceived social support	0.052	921	0.000
Positive coping	0.069	921	0.000
Post-stress growth	0.123	921	0.000
Depressive symptoms	0.099	921	0.000

^*^p < 0.05. ^**^p < 0.01. ^***^p < 0.001. ^a^Kolmogorov–Smirnov test with Lilliefors significance correction.

**Table 3:** Descriptive statistics and correlation matrix of the key variables.

Variables	Mean ± SD	1	2	3	4
1. Perceived social support	59.73 ± 11.53	1			
2. Positive coping	33.75 ± 6.93	0.495^**^	1		
3. Post-stress growth	68.97 ± 10.93	0.490^**^	0.498^**^	1	
4. Depressive symptoms	52.35 ± 10.58	−0.409^**^	−0.443^**^	−0.449^**^	1

^*^p < 0.05. ^**^p < 0.01. ^***^p < 0.001.

### 3.4 Serial mediation effects of coping strategies and post-stress growth

**Figure 1:**
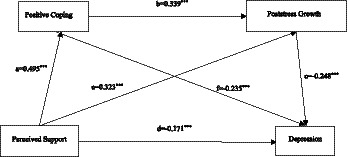
Serial mediation pathways of positive coping strategies and post-stress growth that link perceived social support to depressive symptoms. *p < 0.05. **p < 0.01. ***p < 0.001.

This study investigated the mediating mechanisms that connect perceived social support to depressive symptoms by analyzing the dual mediation pathways that act through positive coping strategies and post-stress growth. Using Hayes’ PROCESS macro (Model 6) with 5,000 bootstrap samples, we evaluated both the individual and sequential indirect effects (Figure 1). Perceived social support emerged as a robust predictor of adaptive functioning, and both direct associations with increased positive coping strategies (*β* = 0.495, *p* < 0.001) and enhanced post-stress growth (*β* = 0.323, *p* < 0.001) were demonstrated. These mediating variables subsequently exerted protective effects against depressive symptoms, where positive coping strategies reduced emotional distress (β = −0.235, *p* < 0.001) and post-stress growth further mitigated depression severity (β = −0.248, *p* < 0.001).

A critical finding emerged from the interrelationship between the mediators: positive coping strategies not only directly alleviated depressive symptoms but also facilitated post-stress growth (β = 0.339, *p* < 0.001), thereby establishing their sequential mediating role. This chained pathway—where social support enhances coping capacity, which in turn promotes growth experiences, which further buffer against depression—accounts for the significant variance in symptom reduction. The total mediation model confirmed the following three viable routes through which social support influences mental health outcomes: the direct effects of coping strategies, the independent effects of post-stress growth, and their synergistic sequential mediation (see Table 4 for detailed effects). These results highlight the cascading psychological benefits of social resources, where supportive environments enable adaptive coping behaviors that, in turn, foster resilience through transformative growth experiences (Table 4).

**Table 4:** Multiple regression analysis of perceived social support, positive coping strategies, post-stress growth, and depressive symptoms.

Model component	Fit indices			Regression coefficients		95% CI
	R	R^2^	F	*β*	*t*	
Positive coping	0.495	0.245	298.054			
Perceived social support				0.495	17.264^***^	0.439 to 0.551
Post-Stress growth	0.572	0.327	223.124			
Perceived social support				0.323	10.363^***^	0.262 to 0.384
Positive coping				0.339	10.867^***^	0.277 to 0.400
Depressive symptoms	0.544	0.285	121.907			
Perceived social support				−0.171	−5.044^***^	−0.238 to −0.105
Positive coping				−0.235	−6.882^***^	−0.302 to −0.168
Post-Stress growth				−0.248	−7.271^***^	−0.314 to −0.181

^*^p < 0.05. ^**^p < 0.01. ^***^p < 0.001.

The mediation hypotheses were tested using a bias-corrected bootstrap procedure with 5,000 resamples. Effects were considered significant when their 95% confidence intervals excluded zero. The analyses revealed three distinct pathways through which perceived social support influences depressive symptoms. The direct pathway accounted for 41.81% of the total effect (β = −0.171, 95% CI [−0.238, −0.105]). The indirect effects operate through two independent mediators and their sequential combination. Positive coping strategies mediated 28.36% of the total effect (β = −0.116, 95% CI [−0.152, −0.081]), whereas post-stress growth independently mediated 19.56% of the total (β = −0.080, 95% CI [−0.110, −0.054]). Crucially, the chained pathway extending through both mediators—where social support enhances adaptive coping, which subsequently promotes growth experiences that reduce depression—explained an additional 10.27% of the total effect (β = −0.042, 95% CI [−0.060, −0.026]). Collectively, these mechanisms accounted for 58.19% of the observed association between social resources and emotional distress, confirming the predominance of the indirect psychosocial processes over direct effects (Table 5).

**Table 5:** Serial mediation effects of positive coping strategies and post-stress growth.

Mediation pathway	Effect	95% CI	Proportion of total effect (%)
Perceived support → Positive coping → Depression	−0.116	−0.152 to −0.081	28.36
Perceived support → Post-stress growth → Depression	−0.080	−0.110 to −0.054	19.56
Perceived support → Positive coping → Post-stress growth → Depression	−0.042	−0.060 to −0.026	10.27
Total mediation effect	−0.238	−0.280 to −0.195	58.19
Direct effect	−0.171	−0.238 to −0.105	41.81
Total effect	−0.409	−0.468 to −0.350	100

^*^p < 0.05. ^**^p < 0.01. ^***^p < 0.001.

To further verify the robustness of the core pathway, this study controlled for variables such as gender, place of origin, and single-child status. The results (Tables 6, 7) indicate that the significance, effect size, and directionality of this pathway (perceived social support → positive coping → post-traumatic growth → depressive symptoms) remained stable and consistent with theoretical predictions without significant alteration. This strongly supports the view that this chained mediation model serves as a unique and robust mechanism explaining the relationships among variables.

**Table 6:** Multiple regression analysis including control variables.

Model component	Fit indices			Regression coefficients		95% CI
Positive coping	0.496	0.246	74.576			
Perceived social support				0.297	17.223***	0.264 to 0.332
Gender				−0.0065	−0.186	−0.075 to 0.062
Place of Origin				0.036	0.768	−0.0561 to 0.128
Only Child				−0.0357	−0.708	−0.056 to 0.128
Post-Stress growth	0.573	0.328	89.429			
Perceived social support				0.203	10.2584***	0.164 to 0.241
Positive coping				0.357	10.8769***	0.293 to 0.421
Gender				−0.028	−0.810	−0.096 to 0.040
Place of origin				−0.024	−0.518	−0.116 to 0.067
Only child				0.048	0.964	−0.050 to 0.147
Depressive symptoms	0.537	0.288	61.665			
Perceived social support				−0.0751	−5.018***	−0.104 to −0.046
Positive coping				−0.172	−6.853***	−0.221 to −0.122
Post-stress growth				−0.172	−7.249***	−0.219 to −0.125
Gender				0.044	1.767	−0.005 to 0.092
Place of origin				−0.015	−0.461	0.081 to 0.050
Only child				0.026	0.719	−0.045 to 0.097

^*^p < 0.05. ^**^p < 0.01. ^***^p < 0.001.

**Table 7:** Serial mediation effects with control variables.

Mediation pathway	Effect	95% CI	Proportion of total effect (%)
Perceived support → Positive coping → Depression	−0.051	−0.067 to −0.036	28.5%
Perceived support → Post-stress growth → Depression	−0.035	−0.048 to −0.023	19.6%
Perceived support → Positive coping → Post-stress growth → Depression	−0.018	−0.026 to −0.012	10.1%
Total mediation effect	−0.104	−0.123 to −0.086	58.1%
Direct effect	−0.075	−0.104 to −0.046	41.9%
Total effect	−0.179	−0.205 to −0.153	100%

^*^p < 0.05. ^**^p < 0.01. ^***^p < 0.001.

## 4 Discussion

Depressive symptomatology represents a prevalent mental health challenge for vocational college students and significantly impedes their psychological well-being and academic functioning. Unlike traditional university cohorts, vocational students are frequently faced with intensified life stressors, including socioeconomic pressures and perceived academic inferiority, that heighten their vulnerability to emotional distress. These unique challenges often manifest as chronic frustrations, self-deprecating cognitions, and learned helplessness, creating fertile ground for depressive episodes (Cao and Zheng, 2023). While the extant research has mapped the depression trajectories in general undergraduate populations (Klettner et al., 2023; Puolakanaho et al., 2023), critical gaps persist regarding vocational students’ psychological ecosystems, particularly the mediating roles played by adaptive coping mechanisms and postadversity growth in the buffering of emotional distress.

This investigation addresses this empirical gap by examining the ways that vocational students’ perceived social support operates through two synergistic pathways—positive coping strategies and post-stress psychological growth—to mitigate depressive symptoms. Our findings delineate a cascading protective model in which social support resources not only directly alleviate emotional distress but also enhance students’ capacity for constructive problem solving. These adaptive coping efforts subsequently foster resilience through transformative growth experiences following adversity, which ultimately disrupts depression trajectories. The identified chained mediation pathway (social support → coping → growth → depression reduction) accounts for more than half of the total protective effect, underscoring the dynamic interplay in mental health preservation that occurs among environmental resources, behavioral activation, and meaning-making processes.

### 4.1 Psychosocial buffering effects of social support on depression

The results revealed a significant negative association between perceived social support and depressive symptoms among vocational college students (*β* = −0.171, *p* < 0.001), which is consistent with the established literature (Chen et al., 2023). This finding aligns with the main-effect model of social support (Chen et al., 2023; Pang et al., 2023; Yang et al., 2023) which posits that individuals who have stronger perceived social support inherently benefit from psychological resource buffering, regardless of their stress exposure. Students reporting limited perceived support may interpret their social environment as being inadequately resourced, thereby experiencing heightened vulnerability to emotional distress in the face of academic or interpersonal challenges. The protective role of social support operates through its capacity to enhance the perception of control over stressors while reducing any maladaptive cognitive appraisals of adversity. Collectively, these mechanisms mitigate depression risk characterized by compounded stressors in a vocational education context. Empirical evidence underscores three critical sources of social support, namely, parental guidance, peer networks, and teacher mentorship, as serving as foundational resources for the maintenance of emotional equilibrium among vocational students (Sun et al., 2020). Parental support provides dual psychological buffers through emotional validation and pragmatic assistance, while peer interactions cultivate belongingness, and teacher–student relationships reinforce academic self-concepts. These multidimensional factors support synergistic effects to disrupt depression pathogenesis.

Notably, gender-specific patterns emerge in support utilization. Male students benefit primarily from stress-buffering effects during crises, whereas female students experience continuous protective effects regardless of their stress exposure (Tanigawa et al., 2011). This divergence suggests the tailoring of intervention approaches, e.g., the developing of crisis-responsive support for male students while concurrently fostering stable support ecosystems for female cohorts.

### 4.2 Mediating function of positive coping strategies

The findings confirm the claim that positive coping strategies significantly mediate the relationship between perceived social support and depressive symptoms among vocational college students. Specifically, higher levels of perceived social support predict a greater adoption of positive coping strategies, which in turn reduces depressive symptoms. This aligns with the social buffering model (Klettner et al., 2023), which posits that social support mitigates the psychological impact of stressors by fostering adaptive responses. Students who perceive stronger support networks are more likely to interpret challenges as being manageable, thereby mobilizing problem-solving strategies rather than avoidance or rumination. Empirical studies have further clarified this mechanism: individuals who report elevated perceived social support often access both tangible resources and emotional reassurance, thereby enabling a proactive approach to stressors (Dai et al., 2023). For example, vocational students who report robust parental or peer support demonstrate an increased use of cognitive restructuring and help-seeking behaviors in the face of academic pressures (Chen et al., 2022). These adaptive strategies disrupt negative emotional cycles by enhancing self-efficacy, which ultimately reduces vulnerability to depressive symptoms. Critically, the mediation pathway identified here underscores the importance of social resources in the shaping of coping behaviors, which is a dynamic of particular relevance for vocational students navigating high-stress educational environments. The capacity of positive coping strategies to mitigate depressive symptoms operates through problem-solving efficacy and emotional regulation. Individuals who employ adaptive coping methods—such as proactive planning and cognitive reframing—demonstrate enhanced stress resolution capacities, which disrupt the cyclic relationship between chronic stress and emotional distress (Areba et al., 2018; Zamanian et al., 2021). This aligns with social cognitive theory’s tenet that depression arises from dynamic person–environment transactions where coping strategies serve as critical moderators of stressor impact (Zhao et al., 2017). The empirical evidence delineates a clear pathway: proactive coping behaviors facilitate concrete problem resolution, thereby reducing both the objective burden of stressors and the subjective emotional toll that they impose (Pelekanakis et al., 2022). For vocational college students, this manifests as a capacity for leveraging perceived social support networks, i.e., the seeking of guidance from mentors, peers, or family, to implement solution-focused strategies during academic or interpersonal crises. Such behaviors not only address any immediate stressors but also reinforce an individual’s self-efficacy beliefs, thereby creating a protective cycle against depressive symptom escalation.

### 4.3 Mediating role of post-stress growth

The results confirm that post-stress growth significantly mediates the relationship between perceived social support and depressive mood among vocational college students. Specifically, higher levels of perceived social support predict greater post-stress growth, which is consistent with findings that demonstrate the role of social support in fostering resilience following adversity (Idås et al., 2019). This mechanism operates through the theoretical framework of self-regulation transfer theory (Benight et al., 2017), where social support equips individuals with both practical resources and cognitive tools for reframing negative experiences. By facilitating access to problem-solving strategies and promoting the positive reappraisal of stressors, social support enables students to extract developmental benefits from challenging situations (Tedeschi and Calhoun, 1996). Notably, the sustaining influence of social support extends beyond initial trauma exposure. Longitudinal evidence indicates that perceived social support maintains posttraumatic growth trajectories over time, serving as a stabilizing factor against emotional deterioration. This enduring effect highlights the dual-phase role played by social resources, in which immediate support buffers acute distress during crises, whereas sustained support networks reinforce the adaptive cognitive patterns that inhibit depression recurrence. These findings underscore the importance of maintaining consistent support systems throughout the academic journeys of vocational student, particularly during transitional periods that are marked by heightened stress exposure. Moreover, social support networks facilitate authentic emotional expression during adversity, enabling individuals to process stressors through constructive cognitive reappraisal (Gök and Çiftçi, 2023). This process is aligned with the observed inverse relationships between post-stress growth and depressive symptoms, where higher levels of growth correspond to reduced emotional distress (Park and Im, 2021). The mediation mechanism involves sequential cognitive–behavioral adjustments, where individuals who appraise stressors through a growth-oriented lens tend to implement targeted coping strategies, gradually developing adaptive cognitive frameworks that enhance emotional regulation and attenuate depressive symptoms. Clinical parallels have emerged in populations facing significant health challenges. Research indicates that many cancer survivors experience posttraumatic growth, which correlates with a reduction in depressive symptoms through strengthened psychological resilience and renewed life prospects (Heidarzadeh et al., 2016). Translating these insights into vocational education contexts and fostering cognitive reframing practices could help students reinterpret academic pressures as catalysts for skill development. Educators can integrate structured reflection exercises that emphasize the extraction of meaning from challenges, thereby promoting post-stress growth trajectories while mitigating depressive outcomes.

### 4.4 Chain-mediating role of positive coping strategies and post-stress growth

Our results confirm that positive coping strategies and post-stress growth sequentially mediate the protective effects of perceived social support against depressive symptoms among vocational students. This chain mediation operates through three interrelated mechanisms: stronger perceived social support predicts the increased use of adaptive coping strategies, which subsequently fosters psychological growth following adversity and ultimately reduces depressive symptoms. These findings align with cross-cultural evidence that demonstrates the coping–growth correlation (Farhadi et al., 2022; An et al., 2013), in which proactive strategies such as cognitive reframing enhance both life meaning (Arslantürk and Öz, 2020) and subjective well-being (Fariddanesh and Rezaei, 2019). Clinical observations have validated this pathway—burn patients employing acceptance-based coping exhibit accelerated rehabilitation engagement and psychological adaptation (Martin et al., 2021), which mirrors the capacity of vocational students to reinterpret stressors as developmental challenges through help-seeking and cognitive restructuring (An et al., 2013; Carr, 2007). Social support systems provide both material resources and emotional scaffolding (Lin, 2016), thereby enabling individuals to transform crises into opportunities for self-discovery while cultivating optimistic mindsets that disrupt depressive cycles. Educators can amplify these protective effects through the application of targeted interventions, such as enhancing student awareness of available support networks, integrating cognitive–behavioral techniques to strengthen adaptive coping, and fostering environments that reward growth-oriented stress reappraisal. Such strategies operationalize the identified mediation sequence in which social resources mobilize coping tools that drive transformative growth, thereby attenuating emotional distress through sustained self-efficacy and purposefulness.

### 4.5 Mechanisms of mediation path differences

This study not only confirms the independent mediating roles of positive coping style and post-stress growth between perceived social support and depressive symptoms among vocational college students, as well as the existence of their serial mediation path, but more importantly, it reveals through effect size decomposition that these three mediation paths exhibit significant differences (see Table 3). Specifically, the sole mediating effect of positive coping style (accounting for 28.36% of the total effect) was the strongest, followed by the sole mediating effect of post-stress growth (accounting for 19.56% of the total effect), while the serial mediation effect of positive coping → post-stress growth (accounting for 10.27% of the total effect) was relatively weaker. This difference may indicate distinct hierarchical roles and temporal dynamics of these pathways in emotional regulation. The stronger effect of positive coping could stem from its nature as a more direct and proximal emotional regulation strategy (Skinner et al., 2003). When vocational students perceive high levels of social support, they tend to immediately employ active strategies (e.g., problem-solving, seeking help) to address stressors (such as academic or interpersonal difficulties). Such proactive regulation alleviates negative emotions (e.g., helplessness, frustration) more rapidly, thereby demonstrating a statistically more significant mediating contribution (Areba et al., 2018).

Although the independent mediating effect of post-stress growth was slightly weaker than that of positive coping, its proportion (nearly 20%) underscores its substantial importance. This pathway likely involves deeper cognitive restructuring and meaning-making processes (Carr, 2007). Perceived social support provides a sense of security and resources, enabling individuals to engage in positive reflection and cognitive adjustment after stress exposure (e.g., altering life perspectives, enhancing self-awareness). The resulting inner strength and positive outlook effectively buffer depressive symptoms (Park and Im, 2021; Boehm-Tabib and Gelkopf, 2021); however, its effects may manifest more slowly or require more stable environmental support.

The relatively weaker serial mediation effect may be related to its reliance on a dual-stage transformation process (Peters et al., 2021; Wan et al., 2022). Perceived social support must first empower individuals to adopt positive coping strategies (first-stage mediation), and the effective application of these strategies (e.g., active problem-solving, seeking information) subsequently fosters cognitive transformation and growth experiences in adversity (second-stage mediation), ultimately reducing depression. This extended chain may imply greater demands on internal resources (e.g., cognitive flexibility, agency) or sustained external support. It may also reflect limitations of the cross-sectional design in capturing long-term processes Farhadi et al., 2022). Nevertheless, its significant existence (10.27% of the total effect) validates that positive coping serves as a crucial bridge to post-stress growth and thereby alleviates depressive symptoms (Zhang et al., 2022a, 2022b), highlighting the necessity of integrating both proactive behavior and cognitive transformation in mental health interventions.

### 4.6 Limitations and future directions

Although this study reveals the chain-mediating mechanism through which perceived social support influences depression in higher vocational students via positive coping styles and post-stress growth, several limitations should be acknowledged:

1. Cross-sectional design constraints: The use of cross-sectional data limits causal inferences. Future research should employ longitudinal tracking or experimental interventions (e.g., social support training programs) to validate dynamic pathways;
2. Limited sample representativeness: Participants were recruited from only three vocational colleges in Guangxi Province, with overrepresentation of rural-origin students (84.4%). Generalizability requires validation across broader geographical regions with balanced urban–rural samples, and comparisons with student groups from different academic levels;
3. Undifferentiated measurement of social support: The global construct of perceived social support was examined without distinguishing subdimensions (e.g., family, friends, teacher support). Future investigations should explore differential effects of support sources to inform targeted interventions;
4. Uncontrolled confounding variables: Potential factors influencing both post-stress growth and depression (e.g., individual trauma history, socioeconomic status) were not controlled. Subsequent studies should incorporate relevant covariates to enhance model rigor;
5. Self-report bias: Exclusive reliance on self-reported questionnaires risks social desirability bias. Multimethod approaches (e.g., interviews, behavioral observations) are recommended to improve validity.

Future research addressing these limitations will advance mechanistic understanding and practical applications.

## 5 Conclusion

This study took vocational college students as the research objects to explore the mediating mechanisms of positive coping strategies and post-stress growth between the factors of perceived social support and the depressive mood of vocational college students. The study reveals a significant positive correlation among perceived social support, positive coping strategies, and post-stress growth, and all three variables are significantly negatively correlated with the depressive mood of vocational college students. Perceived social support not only directly and negatively predicts the depressive mood of vocational college students but also influences it through both the independent mediating effects of positive coping strategies and post-stress growth and the chain mediating effect of positive coping strategies on post-stress growth. These findings are helpful for understanding the mechanisms underlying the depressive mood of vocational college students and confirming that perceived social support, positive coping strategies, and post-stress growth all serve as important factors in combating depressive mood; that is, these factors help reduce the depressive mood of vocational college students.

## Data Availability

The raw data supporting the conclusions of this article will be made available by the authors, without undue reservation.
